# Characterization of the complete mitochondrial genome of *Loxocephala perpunctata* (Hemiptera: Eurybrachidae)

**DOI:** 10.1080/23802359.2022.2080013

**Published:** 2022-06-10

**Authors:** Shi-Yan Xu, Xiang-Sheng Chen

**Affiliations:** a College of Life Science, Guizhou Normal University, Guiyang, China; b Institute of Entomology, Guizhou University, Guiyang, China

**Keywords:** Mitochondrial genome, Hemiptera, Eurybrachidae

## Abstract

In this study, we sequenced and annotated the complete mitochondrial genome of *Loxocephala perpunctata* (Jacobi, 1944) (Hemiptera: Eurybrachidae). The mitogenome of *L. perpunctata* is 15,017 bp long and includes 37 genes and a large control region. Consisting of 13 protein-coding genes, 22 tRNA genes, 2 rRNA genes, and a D-loop. All protein-coding genes have the usual ATN start codons, except for *ND5*, which uses the noncanonical codon GTG. 22 tRNAs, the length ranging from 59 to 69 bp, having the clover-leaf structure except for the dihydrouridine (DHU) arm of trnS2 forming a simple loop.

The Eurybrachidae is widely distributed in the world and established by Stål (1862), which includes 41 genera and 202 species (Bourgoin et al. 2020). Eurybrachidae have been so far described all over the world. Like other hemipteran insects, the phytophagous insects extract plant sap using their sucking and piercing mouthparts, causing abnormal proliferation of plant cells, affecting plant growth and development, spreading plant viral diseases, and leading to severe damage to grain production. But more, most studies still focus on the description of new species, and no molecular data were used for the phylogeny of Eurybrachidae. *Loxocephala* was erected by Schaum (1850). Currently, the genus *Loxocephala* has been described as 13 described species and one subspecies. Here, we determine the mitogenome sequence of *Loxocephala perpunctata* to reveal the evolution of the Eurybrachidae.

The samples were collected from Weining County, Caohai City, Guizhou Province, China in August. 2018 (104°12′ E, 26°49′ N). A specimen was deposited in the Institute of Entomology, Guizhou University, China (No. GZEU-01, Shiyan Xu, syxu1020@126.com). A total genomic DNA was extracted from *L. perpunctata* with TIANGEN Genomic DNA Extraction Kit (TIANGE, Beijing, China), according to the manufacturer’s instructions. The complete mitogenome sequence of *L. perpunctata* is based on next-generation sequencing. An Illumina ReSeq library was prepared with an average insert size of 400 bp and sequenced using the Illumina NovaSeq6000 platform with 150 bp paired-end (Berry Genomics, Beijing, China). The complete mitogenome sequence was assembled using NOVOplasty v2.7.2 (Dierckxsens et al. 2017) with K-mer value, and annotated by MitoZ v2.4 (Meng et al. 2019) with default settings. Genomic annotation was calibrated using MITOS2 (Bernt et al. 2013) and Geneious Prime v2020.2.4 (Kearse et al. 2012).

The complete mitochondrial genome of *L. perpunctata* (GenBank accession number: MW848343) was a closed circle molecule of 15,017 bp in length, consisting of 37 encoding genes (13 PCGs, 22 tRNA genes, and 2 rRNA genes) and one control region. The whole base composition of the J-strand is as the following: 47.3% of A, 7.5% of G, 11.7% of C, and 33.5% of T, with a high representation of nucleotides AT. Typically, all protein-coding genes had the usual ATN start codons (ATT for *ND2*; ATG for *COI*, *ATP6*, *COIII*, *ND4*, *ND4L*, *CytB*, and *ND1*; ATA for *COII*, *ATP8*, *ND3*, and *ND6*), except for *ND5*, which used noncanonical codon GTG. All protein-coding genes use the TAA or T as stop codons, except for *ND3*, which uses the stop codon TAG. 22 tRNA genes, ranging from 59 to 69 bp, have a typical cloverleaf structure except for the dihydrouridine (DHU) arm of trnS2 which forms a simple loop.

The phylogenetic tree was constructed by maximum-likelihood (ML) analysis, based on the dataset of the 13 protein-coding genes and two rRNA genes from 12 species of Fulgoroidea. In the ML tree, Fulgoroidea is divided into two groups: Delphacidae and other families of Fulgoroidea, Delphacidae taxa feed near the ground, while members of the remaining families feed higher on their host plants. The results support Lophopidae and Eurybrachidae, Achilidae and Fulgoridae, Issidae and Ricaniidae are sisters in the relationship between these families (Figure 1).

**Figure 1.**
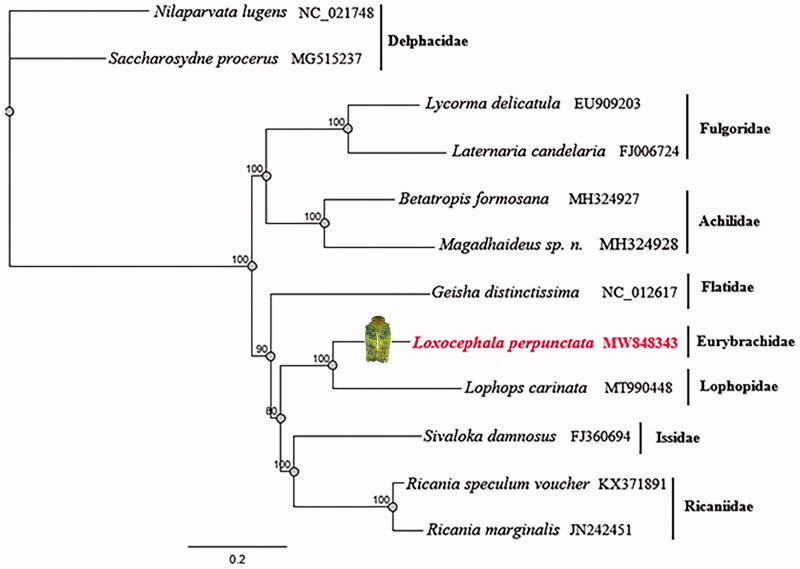
Maximum-likelihood phylogenetic tree inferred from 13 protein-coding genes and 2 rRNA genes.

## Author contributions

Chen Xiangsheng designed the research study and obtained the funding. Xu Shiyan performed laboratory work and bioinformatics analyses.

## Data Availability

The data that support the finding of this study are openly available in NCBI at https://www.ncbi.nlm.nih.gov, reference number [MW848343]. The associated BioProject, Bio-Sample, and SRA numbers are PRJNA758227, SAMN21155277, and SRR15675949, respectively.
